# Polycythemia Vera Presenting as Cardiac Arrest: Novel Management Strategies

**DOI:** 10.1155/2019/9656387

**Published:** 2019-01-22

**Authors:** Mark I. Davis, Brian K. Courtney, Gideon Cohen, Stephanie Poon, Mina Madan

**Affiliations:** ^1^Division of Cardiology, Department of Medicine, Sunnybrook Health Sciences Centre, University of Toronto, Toronto, Canada; ^2^Division of Cardiac Surgery, Department of Surgery, Sunnybrook Health Sciences Centre, University of Toronto, Toronto, Canada

## Abstract

Acute coronary syndromes (ACS) usually occur in patients with multiple cardiac risk factors. In young adults, drug use and hypercoagulable states are common causes for ACS presentations. We report a case of a man in his early 30s who was diagnosed with polycythemia vera (PV) and had a cardiac arrest due to an anterolateral ST elevation myocardial infarction. We discuss his unique management and review the evidence on the management of arterial thromboembolism in PV patients.

## 1. Introduction

Acute coronary syndrome presentations in young adults are often unrelated to atherosclerosis and instead are commonly due to drug use (i.e., cocaine), underlying hypercoagulable states, and vascular abnormalities such as fibromuscular dysplasia. Additionally, myeloproliferative disorders such as polycythemia vera (PV) can lead to acute arterial thrombotic occlusions. Here, we present a case of a patient with PV, who had massive coronary artery thrombosis presenting with sudden cardiac arrest. Although there have been many similar case reports [1–6], none have described an ST elevation myocardial infarction with ventricular fibrillation arrest presentation nor the extent of arterial thrombosis observed in this patient and its unique management.

## 2. Case Report

A male in his early 30s had a witnessed cardiac arrest shortly after an emotionally stressful event. Bystander CPR was initiated immediately. When emergency medical services arrived, the presenting rhythm was ventricular fibrillation and 1 shock was delivered. He was intubated at the scene. Standard advanced cardiac life support was continued en route to the nearest emergency department. In total, four cycles of CPR, epinephrine, and defibrillation were given prior to the return of spontaneous circulation, obtained upon the patient's arrival at a community hospital. We were urgently contacted by the community hospital because his initial ECG was consistent with left bundle branch block morphology but then progressed on serial ECGs with significant ST elevations noted in leads I, AVL, and V4-V6 and ST depressions in leads III and aVF. The patient was transferred emergently to our institution for emergency coronary angiography in the setting of ST elevation myocardial infarction (STEMI). The patient's past medical history is significant for a stroke at age 7 without any residual deficits and hypertension. He was recently diagnosed with polycythemia vera (Janus kinase 2 (JAK2) positive) and was prescribed enteric-coated ASA 81 mg daily and hydroxyurea. The patient had previously been undergoing regular phlebotomy at our institution but had not attended these appointments over the last two months. It was unclear whether he was taking any medications at the time of presentation. Initial bloodwork showed the following: hemoglobin 184 g/L, hemotocrit 0.59, platelet count 1072 × 10^9^/L, leukocytes 38.8 × 10^9^/L, creatinine 142 *μ*mol/L, hsTroponin 506 ng/L (peak 67322 ng/L) and CK 980 U/L (peak 9013 U/L), pH 7.12, and lactate was 9 mmol/L.

**Figure 1:**
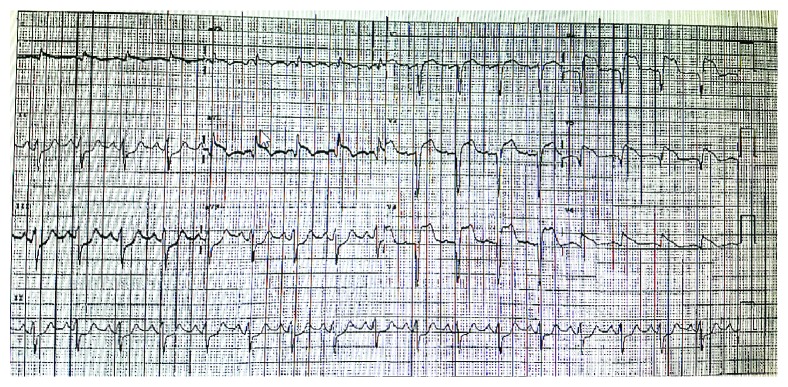
Presenting ECG at our institution: significant anterior and lateral ST elevations along with anterior and lateral Q waves. Based on this ECG as well as his extremis presentation, he was brought emergently to the cardiac catheterization lab.

**Figure 2:**
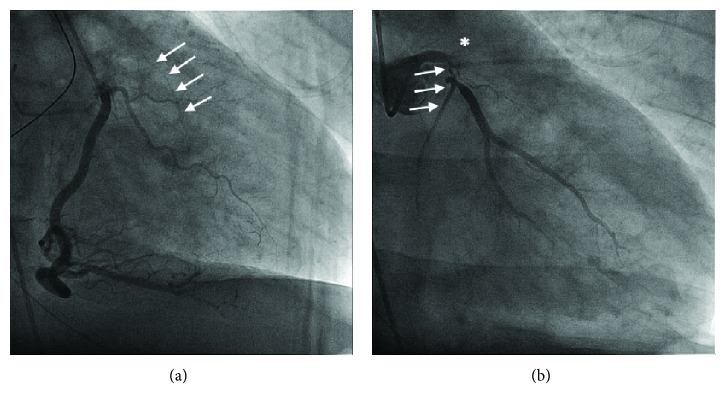
(a). RAO projection demonstrating a normal and patent RCA and left-sided collaterals (white arrows). See Supplementary Video 1 for CINE. (b). RAO caudal projection of LCA. The LAD is occluded (^∗^), and proximal LCX has a long 90% proximal stenosis (white arrows) and fills poorly after a large first obtuse marginal branch. See Supplementary Video 2 for CINE.

**Figure 3:**
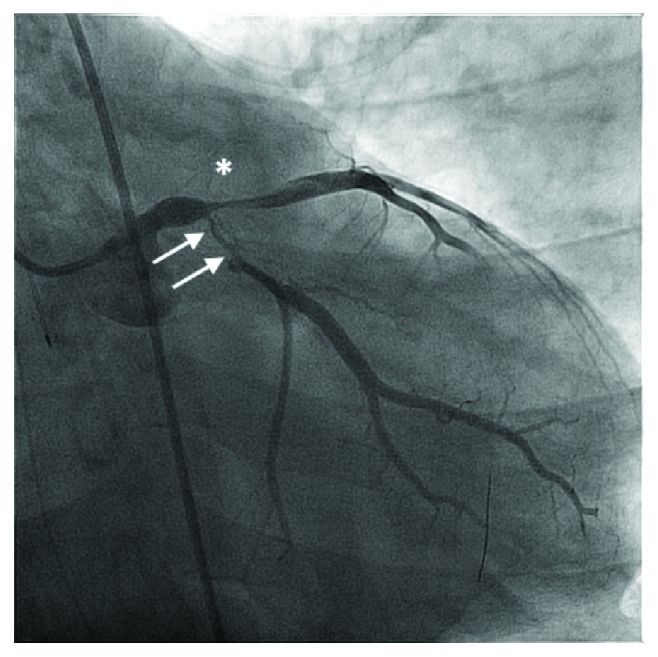
RAO caudal projection after initial ballooning of the LAD and LCX: extensive thrombus burden is noted in the LAD (^∗^) and a 90% proximal LCX lesion (white arrows). See Supplementary Video 3 for CINE.

Upon arrival at our institution, he was in cardiogenic shock with a blood pressure (BP) of 95/80 mmHg and a heart rate (HR) of 110 bpm on 2.5 mcg/kg/min of intravenous (IV) dopamine, 1 mg/min IV amiodarone, 20 mcg/kg/min IV propofol, and intermittent IV boluses of 50 mg rocuronium. He also received 120 mg of IV furosemide. IV norepinephrine was initiated at 10 mcg/min, and dopamine was discontinued. His ECG showed an anterolateral STEMI (Figure 1). ECASA 160 mg and ticagrelor 180 mg were given to the patient via the NG tube, and 4000 units of IV heparin was administered. He was brought urgently to the cardiac catheterization laboratory. Selective coronary angiography demonstrated a normal right coronary artery with collaterals to the left circumflex artery (LCX), complete occlusion of his left anterior descending artery (LAD), and a long 90% lesion of the proximal LCX (Figures 2(a) and 2(b), Supplementary Videos 1 and 2). The left ventricular end diastolic pressure was 30 mmHg. As he was on a significant amount of vasoactive medications for hemodynamic support, we did not believe it was appropriate to administer intra-arterial nitroglycerin at this time, nor did we feel this would lead to any change in his clinical management, as there was clear thrombus in the LM, LAD, and LCX. His oxygen saturation was 95% on 100% FiO_2_, but his BP was 84/68 and HR 118 bpm, despite being on 15 mcg/min of IV norepinephrine. Based on the patient's clinical status and angiographic findings, an 8 French 40 cc intra-aortic balloon pump (IABP, Arrow® International, Teleflex Medical, Athlone, Ireland) was inserted into the right femoral artery followed by the administration of additional 7,000 IU (patient's weight was 110 kg and had already received 4000 units preprocedure) of intra-arterial heparin. We then proceeded with emergency percutaneous coronary intervention (PCI) on the LAD and LCX. There was a significant amount of thrombus in the LAD and LCX (Figure 3, Supplementary Video 3); and thus, 6 passes with a 6 French Export Advance™ Aspiration Catheter (Medtronic Inc., Minneapolis, MN) were performed. Soon after thrombectomy was completed, the patient experienced recurrent thrombosis of the left coronary system. Repeat thrombectomy was performed; however, the thrombectomy catheter itself became clotted.

**Figure 4:**
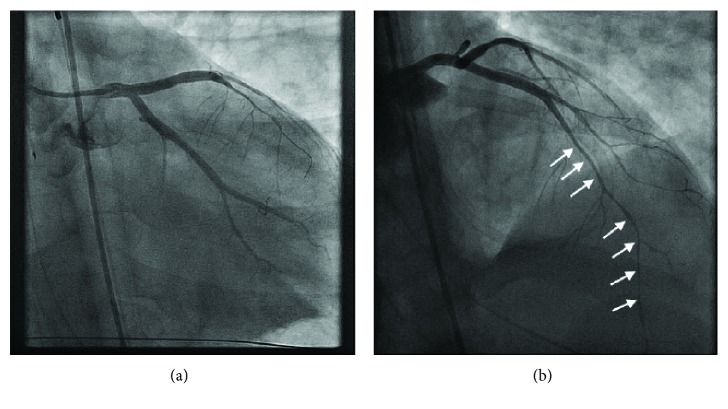
(a). RAO caudal projection. After PCI, the left main artery, LAD, and LCX are patent. See Supplementary Video 4 for CINE. (b). Diffuse distant vasoconstriction of the coronary arteries (white arrows) following PCI due to high doses of IV norepinephrine, epinephrine, and vasopressin. See Supplementary Video 5 for CINE.

To combat the extensive thrombus formation in the setting of PV, we performed intraprocedural phlebotomy. A total of 550 mL of blood was phlebotomized, and two boluses of intracoronary eptifibatide were given. Despite this fact, there was a significant amount of recurrent thrombus formation and a second dose of intra-arterial heparin at 100 IU/kg was given for a total of 21,000 IU of IV unfractionated heparin which was administered for the procedure. Although an ACT was not drawn during the case, at the end of the case, it was 250 seconds. Eventually, the de novo coronary thrombosis stopped, and complex bifurcation stenting of the LAD, left main artery, and LCX could be performed using 2 drug-eluting stents and a bifurcation minicrush technique (Figure 4, Supplementary Videos 4 and 5). Despite these efforts, he remained in cardiogenic shock (BP 101/57 mmHg, HR 107 bpm) while on significant hemodynamic support (IABP at 1 : 1 cycle, IV norepinephrine 20 mcg/min, IV vasopressin 0.04 units/min, and IV epinephrine 20 mcg/min). The advanced heart failure and cardiac surgical teams were consulted, and the decision was made to bring the patient directly to the operating room for an emergency left ventricular assist device (LVAD-CentriMag, Thoratec Corporation, Pleasanton, CA), which the teams felt to be more appropriate than an extracorporeal membrane oxygenation circuit. Additionally, 1 g of hydroxyurea was given at the end of the procedure via an oral gastric tube following consultation with hematology for the acute treatment of PV.

His postoperative course was complicated by left-sided weakness and radiologic evidence of a new stroke (right occipital parietal subacute infarct with hemorrhagic transformation along with multiple small subacute infarcts in the right frontal, left frontal, and left parietal occipital regions), pneumonia, and a slow wean from the LVAD-CentriMag. Postprocedure imaging demonstrated evidence of significant mitral regurgitation secondary to complete flail of the anterior mitral valve leaflet with chordal rupture, which may have occurred following the emergency insertion of the CentriMag device. The patient underwent an Alfieri stitch procedure on LVAD removal, to reduce the degree of mitral regurgitation from severe to moderate. The patient eventually made significant neurological recovery and was discharged from the hospital to an inpatient cardiac rehabilitation facility, 38 days after admission to our institution. His ejection fraction at discharge was 25-35% with moderate mitral regurgitation.

After discharge, the patient had a single chamber ICD inserted due to the persistence of severe LV dysfunction with a left ventricular ejection fraction of 20-25% as well as moderate-to-severe RV dysfunction. The distal half of his LV, as well as the septum, was thinned and akinetic. Within 3 months after discharge, he was readmitted to the hospital with right-sided heart failure symptoms and weight gain. Investigations revealed portal vein thrombosis and right-sided heart failure as the causes. He has required multiple dose adjustments of his cardiac medications due to development of cardiorenal syndrome. At 1 year post MI, his ejection fraction remained <30% with anterior wall akinesis, aneurysmal apex, and moderate to severe mitral regurgitation. He continues to be followed by cardiology, a dedicated heart failure clinic, respirology, neurology, haematology, and nephrology.

## 3. Discussion

Polycythemia vera is a type of chronic myeloproliferative neoplasm. The diagnosis is based on the World Health Organization criteria [7], requiring all three majors or the first major and 1 minor criterion (major criteria: (1) hemoglobin > 165 g/L in men and >160 g/L in women or hematocrit > 49% in men and >48% in women or increased red cell mass, (2) hypercellular bone marrow with trilineage growth, and (3) presence of a JAK2 mutation; minor criteria: hypercellular bone marrow with trilineage growth, low serum erythropoietin levels, and endogenous erythroid colony formation). Clinical symptoms include pruritus, splenomegaly, vasomotor symptoms, and arterial and venous thrombosis. The JAK2 mutation is 95 to 100% sensitive in the diagnosis of PV. Our patient had both the major criteria at the time of diagnosis and has had evidence of arterial thromboembolism both on this current presentation (coronary and cerebrovascular) as well as when he was a child (cerebrovascular). We learned from the patient's hematologist that he was noncompliant with therapy, despite being prescribed cytoreductive therapy (hydroxyurea). Hydroxyurea has been shown to significantly reduce recurrent thrombotic complications such as stroke and myocardial infarction [8]. The incidence of thrombotic complications in PV patients ranges from 13 to 60%. The presence of traditional cardiac risk factors further increases the risk of thrombosis, especially coronary artery thrombosis [9]. Our patient had hypertension and was an active smoker, satisfying the above criteria.

In patients with PV, arterial thrombi form due to platelet activation, adhesion, and aggregation [10]. Proposed mechanisms of thrombogenesis are multifactorial but involve increased shear stress due to high hematocrit leading to vessel wall inflammation and closer contact of platelets to the endothelium leading to platelet activation. The use of intracoronary eptifibatide or abciximab inhibits platelet aggregation by reversibly blocking the GP IIb/IIIa receptor, decreasing fibrinogen and von Willebrand factor-mediated platelet activation [11]. Intracoronary eptifibatide doses can be up to 1000 times that delivered intravenously and can disaggregate the thrombus itself [12]. In the case of our patient, initially, there was recurrent thrombosis and the use of intracoronary eptifibatide may have decreased further thrombus formation, allowing the visualization of the coronary anatomy and PCI to be performed. Previously, a meta-analysis comparing the use of intracoronary to intravenous abciximab demonstrated a mortality benefit of intracoronary abciximab in primary PCI patients, although more recent data suggest no difference in myocardial damage with intracoronary or intravenous GP IIb/IIIa inhibitors [13, 14]. The subsequent INFUSE-AMI trial showed that among early presenting anterior STEMI patients, infarct size is decreased with the use of intracoronary abciximab but not manual aspiration thrombectomy [11]. In our patient, high-dose heparin was used synergistically with eptifibatide to block the coagulation cascade and prevent further intracoronary thrombus formation. To our knowledge, no other case reports have reported double bolus IV unfractionated heparin during primary PCI when there is such extensive thrombus burden. Although our ACT was therapeutic, we believed that in this young male, the benefit of extra heparin outweighed the risk of a potential bleed, and without preventing further thrombus formation, he likely would have died intraprocedurally. As the heparin and eptifibatide took effect, de novo thrombus formation decreased and PCI to the LAD and the LCX was possible. We do not feel that the intraprocedural heparin contributed to the hemorrhagic conversion of his thrombotic stroke, as the procedure and stoke were separated by multiple days and the heparin would have already been cleared from his system. He still would have had to have some heparin on board due to the CentriMag, and this could have increased the risk of hemorrhagic conversion.

In situations where the pathology is unclear, both IVUS and OCT can be helpful to determine pathology and possibly change management [15, 16]. It was clear that the LAD and LCX were occluded with thrombus, which is known to occur in patients with polycythemia vera without underlying atherosclerosis. As seen in the RCA injections, there was some collateral flow to the left system, although collaterals can form quite acutely and do not suggest chronic atherosclerotic disease in this patient [17]. Since this patient was hemodynamically unstable, the goal was to reestablish blood flow, irrespective of the underlying pathology; thus, no adjunctive imaging modalities were used. Furthermore, as we were unable to establish any significant flow down the coronary arteries until stent deployment, there would not have been enough opacification with contrast for OCT to be beneficial.

Prior publications have reported intraprocedural phlebotomy to combat thrombus formation, although they were not known to us at the time [3]. There are no guidelines addressing the acute management of arterial thromboembolism in patients with PV, especially in those with extremis presentations like our patient. The decision for phlebotomy as well as NG hydroxyurea was made empirically by the treatment team to try all means possible to preserve cardiac function and life. Our goal was to acutely reduce the hematocrit to <45%. Indeed, a prior study of non-PV patients which stratified STEMI patients based on hematocrit levels (anemia with hematocrit < 36% for women and <39% for men, normal hematocrit, or erythrocytosis with hematocrit > 46% for women and >47% for men) showed that although the one-month mortality was highest in patients with anemia, the odds ratio for mortality in patients with erythrocytosis was 4.3 [18]. Furthermore, PV treatment guidelines suggest targeting a hematocrit < 45% in PV patients to prevent arterial thromboembolism through phlebotomy and the use of cytoreductive therapy such as hydroxyurea [19, 20]. Although we used these measures to treat his PV acutely, these measures were maintained throughout his subsequent hospital stay. It is unlikely that randomized controlled trials would be able to be conducted with a sufficient number of patients to determine the optimal treatment method for PV patients with massive arterial thromboembolism presenting in an extremis fashion as the patient in our case, and we are acknowledging there is no evidence to support our treatment method.

The management of high intracoronary thrombus burden is difficult, and there are no guidelines regarding this management. Strategies to deal with high intracoronary thrombus burden include the use of adjunctive thrombectomy, intracoronary GP IIb/IIIa inhibitors, and/or intracoronary thrombolysis with PCI if immediate intervention is required, usually due to poor flow or an occluded vessel. But if there is TIMI 3 flow, another approach includes IV heparin and the use of IV GP IIb/IIla inhibitors to allow for clot dissolution and then PCI later. Although the most recent ACC/AHA STEMI guidelines [21] have decreased the use of mechanical thrombus aspiration to Class 2b, for selective use, we do believe our patient was appropriate for aspiration thrombectomy. The ESC STEMI guidelines, [22] also recommended against routine thrombus aspiration, do suggest that thrombus aspiration may be considered in patients with large residual thrombus after wire and balloon attempts to open the vessel (which our patient would fall under) [23]. Although we agree that thrombectomy should not be used in all situations, for this patient, both his LAD and LCX were completely occluded by thrombus and he was in cardiogenic shock. These patients were not included in most of the thrombectomy trials [11, 24], or the number of cardiogenic shock patients was not identified for subgroup analysis [25, 26].

There are no large-scale trials that look at the use of intracoronary thrombolytics but only small single-centre trials that have shown positive results for thrombus dissolution after the use of thrombectomy and/or intracoronary GP IIb/IIIa inhibitors. The DISSOLUTION trial [27], which included approximately 100 patients, with 50 in each arm, showed decreased thrombus burden but no measurable change in MACE. The same was the case for the even smaller ICE T TIMI 49 study, which had 10 patients in each arm and once again showed that there was a decrease in thrombus burden but was not powered for a difference in MACE. Both studies demonstrated safety of IC thrombolytics with minimal to no bleeding events. The dosage used was between 1/5 and 1/3 of the systemic dose. At this dosage, there were no significant bleeding events. In these two small studies and a few other case reports, there seemed to be successful establishment of TIMI 3 flow after other standard PCI techniques failed without significant morbidity or mortality. The best approach in this situation would be the use of a microcatheter to allow for directed thrombolytic injection and not injection through the guide catheter which would result in significant “spillage” and a more systemic effect.

PV patients who undergo PCI are left with having a higher than normal risk of stent thrombosis, although the true risk is unknown, nor do any guidelines exist on the antiplatelet regimen that should be followed. Logically, using more potent P2Y12 inhibitors such as ticagrelor or prasugrel in combination with ASA would lead to a lower stent thrombosis risk than that of clopidogrel. No data exists on the use of anticoagulants in this scenario. Given the propensity for stent thrombosis in these patients, we agree that if the patient can be adequately treated without stent insertion, this would be preferable, but if unavoidable, dual antiplatelet therapy with aspirin and ticagrelor/prasugrel, in addition to hydroxyurea and phlebotomy, would likely result in the least long-term morbidity/mortality and the lowest risk of stent thrombosis.

## 4. Conclusion

Here, we presented a case of a young male with polycythemia vera who was noncompliant with treatment and who presented with sudden cardiac arrest due to massive and recurrent coronary thrombosis. Our unique treatment included a multipronged approach employing thrombectomy, acute phlebotomy, double-bolus intracoronary eptifibatide, and double-dose IV unfractionated heparin, allowing thrombus removal and prevention of recurrent coronary thrombosis. The patient eventually required temporary LVAD placement until his cardiac status improved and he made a relatively complete neurological recovery over 5 weeks from his initial presentation. This case highlights the importance of maintenance therapy among patients with polycythemia vera and the extremis presentation they can have with arterial thrombosis.

## Supplementary Materials

Supplementary 1 Supplementary Video 1: RAO projection of the RCA demonstrating collaterals to the left system. Cinepak Codec.

Supplementary 2 Supplementary Video 2: RAO caudal projection of the LCA demonstrating an occluded LAD and significant stenosis of the proximal LCX. Cinepak Codec.

Supplementary 3 Supplementary Video 3: RAO caudal projection of the LAD and LCX post initial balloon dilation of the LAD. Extensive thrombus burden is seen. Cinepak Codec.

Supplementary 4 Supplementary Video 4: RAO caudal projection of the LAD and LCX post bifurcation stenting of the LAD, LM and LCX. The proximal vessels are patent with distal vessel constriction due to high doses of vasoconstrictors. Cinepak Codec.

Supplementary 5 Supplementary Video 5: RAO cranial projection of the LAD demonstrating a patent vessel with distal vessel vasoconstriction from high doses of norepinephrine, vasopressin, and epinephrine. Cinepak Codec.
